# Ploidy Distribution of the Harmful Bloom Forming Macroalgae *Ulva* spp. in Narragansett Bay, Rhode Island, USA, Using Flow Cytometry Methods

**DOI:** 10.1371/journal.pone.0149182

**Published:** 2016-02-26

**Authors:** Elaine E. Potter, Carol S. Thornber, John-David Swanson, Malcolm McFarland

**Affiliations:** 1 Department of Biological Sciences, University of Rhode Island, Kingston, Rhode Island, United States of America; 2 Department of Biology and Biomedical Sciences, Salve Regina University, Newport, Rhode Island, United States of America; 3 Graduate School of Oceanography, University of Rhode Island, Narragansett, Rhode Island, United States of America; 4 Harbor Branch Oceanographic Institute, Florida Atlantic University, Fort Pierce, Florida, United States of America; Stony Brook University, UNITED STATES

## Abstract

Macroalgal blooms occur worldwide and have the potential to cause severe ecological and economic damage. Narragansett Bay, RI is a eutrophic system that experiences summer macroalgal blooms composed mostly of *Ulva compressa* and *Ulva rigida*, which have biphasic life cycles with separate haploid and diploid phases. In this study, we used flow cytometry to assess ploidy levels of *U*. *compressa* and *U*. *rigida* populations from five sites in Narragansett Bay, RI, USA, to assess the relative contribution of both phases to bloom formation. Both haploid gametophytes and diploid sporophytes were present for both species. Sites ranged from a relative overabundance of gametophytes to a relative overabundance of sporophytes, compared to the null model prediction of √2 gametophytes: 1 sporophyte. We found significant differences in cell area between ploidy levels for each species, with sporophyte cells significantly larger than gametophyte cells in *U*. *compressa* and *U*. *rigida*. We found no differences in relative growth rate between ploidy levels for each species. Our results indicate the presence of both phases of each of the two dominant bloom forming species throughout the bloom season, and represent one of the first studies of *in situ Ulva* life cycle dynamics.

## Introduction

Macroalgal blooms typically consist of large accumulations of ephemeral macroalgal biomass. These blooms occur worldwide, often in shallow areas with relatively low water mixing that are affected by coastal eutrophication, and they have the potential to cause severe ecological and economic damage [1–3]. The largest documented bloom on record occurred four weeks before the 2008 Beijing Olympics, with a bloom of an estimated 20 million tons of *Ulva prolifera* in the Yellow Sea near Qingdao, China [4–6]. The costs of clean up for the bloom were estimated at 30.8 million US dollars, not including losses to aquaculture and tourism [4]. The ecological effects of macroalgal blooms are often far-reaching and indirect; algal blooms negatively affect seagrass beds, sessile invertebrates and perennial algae [7–9]. Large blooms can create hypoxic environments that contribute to mass fish and invertebrate die-offs [10, 11] and hydrogen sulfide from decaying algal mats can cause symptoms such as difficulty breathing and nausea in humans [12]. Large macroalgal blooms decrease light attenuation and shade seagrass beds and benthic perennial algae [13]. Blooms have increased worldwide over the years in frequency and intensity [14–16].

The green macroalgal genus *Ulva* forms large and dense sheets, a phenomenon known as green tides, and can proliferate by asexual (e.g., fragmentation, spore formation) [5, 17, 18] and sexual reproduction [19]. Green tides include many genera of green algae, such as *Chaetomorpha*, and affect at least 37 countries worldwide [20, 21]. *Ulva* is one of the most common macroalgal bloom-forming genera present in green tides and is the focus of this study.

**Fig 1:**
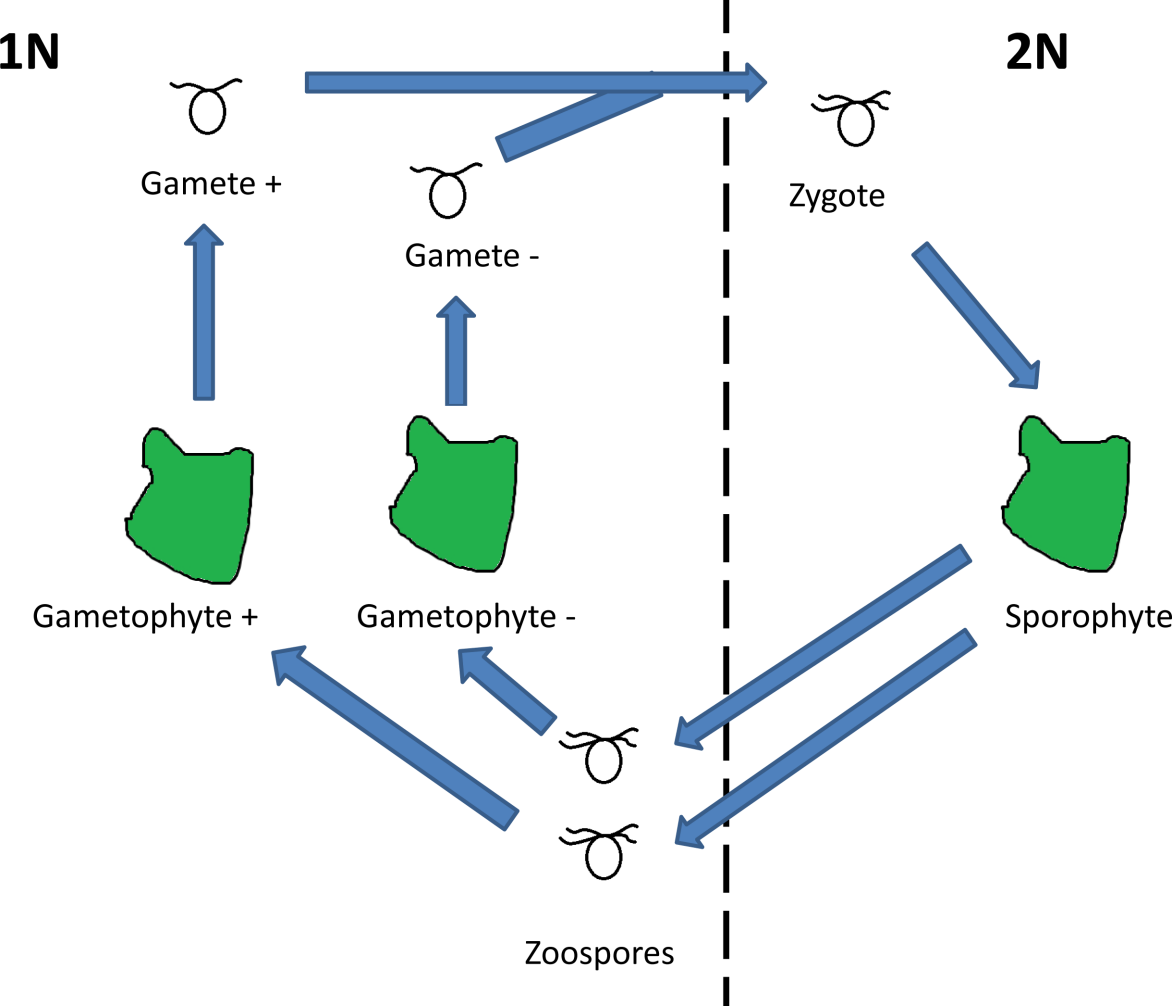
Isomorphic biphasic life cycle of *Ulva*. *Ulva* cycles between two morphologically similar multicellular adult phases, a haploid gametophyte and a diploid sporophyte. Diploid sporophytes produce haploid zoospores that develop into gametophytes. Haploid gametophytes produce haploid gametes. When a “+” and “–” gamete fuse a zygote is formed, which develops into a diploid sporophyte.

Like many marine algae, *Ulva* has a biphasic life cycle consisting of an alternation between two free-living forms, a haploid phase (1N, gametophyte) and a diploid phase (2N, sporophyte; Fig 1). These phases of *Ulva* are isomorphic, meaning that the gametophyte and sporophyte are morphologically similar and cannot be visually distinguished in the field. If the phases are ecologically equivalent, we expect a ratio of √2 gametophytes: 1 sporophyte at equilibrium [22]. This deviation from a 1 to 1 ratio is because *Ulva* is dioecious; each spore produced by the diploid sporophyte can potentially result in a haploid gametophyte, but only the female gametophyte can produce a sporophyte, resulting in a relative overabundance of adult gametophytes. The √2 gametophytes: 1 sporophyte ratio should be observed as long as there are no differences in mortality and fecundity between the two phases. For isomorphic algal species, however, a wide range of distributions of ploidy ratio in have been documented in the field [23].

There are few published studies on the *in situ* life cycle dynamics of *Ulva*; Hiraoka and Yoshida [19] found a non-seasonal alternating dominance of the two phases for *U*. *pertusa*, Alström-Rapaport and colleagues [24] and Pringle [25] found a seasonal shift, with an increase of the proportion of gametophytes during the summer months in *U*. *(prior Enteromorpha) intestinalis*, although sporophytes were always more abundant. This lack of a broader understanding of *Ulva* life cycle dynamics may be due to the difficulty of discerning between isomorphic phases; however, ploidy can be rapidly determined using flow cytometry [26–28]. Flow cytometry quantitatively analyzes the DNA content of nuclei in a suspended solution and can allow for a convenient, fast, and reliable method for determining ploidy.

Although isomorphic sporophytes and gametophytes appear identical, they can occupy different ecological niches [29–31]. For example, one phase may be responsible for forming blooms, while the other may occur during non-bloom forming months, although data on these dynamics are relatively unknown [32, 33]. In addition, the two phases may vary in growth rates, temperature optima, or susceptibility to herbivores [34]. Similarly, phases could vary in their response to environmental variables such as temperature and nutrients [29, 35]. If there are ecological differences between *Ulva* gametophytes and sporophytes, the distribution of life history phases will be partially dependent upon the physical and biological factors of the system.

Some advantages for sporophytes include the ability to mask deleterious mutations [36] resulting in increased genetic diversity, the accumulation of mutations at twice the rate of gametophytes [37], and extra flexible alleles that contribute to faster adaptation by evolving to serve new functions [38]. Advantages for gametophytes include the immediate elimination of deleterious mutations, faster evolution due to strong selection on beneficial alleles [39], and lower nutrient requirements [40].

We investigated life cycle dynamics in the bloom-forming macroalgae *Ulva compressa* L. and *Ulva rigida* C. Agardh, which are common in summer macroalgal blooms in the estuarine system of Narragansett Bay, Rhode Island [41, 42]. Macroalgal densities (comprised mostly of *Ulva*) peak in the summertime and vary significantly across sites, seasons, and years [43–45].

Our research focuses on four central questions regarding the life cycles and biology of *U*. *compressa* and *U*. *rigida*. Firstly, what is the relative abundance of sporophytes and gametophytes of both species? Secondly, how do these relative abundances correlate with physical and biological factors? Thirdly, do the phases have different growth rates, and lastly, do the phases have cells of different sizes? We interpret our data in the context of macroalgal bloom dynamics and the impacts of environmental variables in structuring bloom formation.

## Materials and Methods

### Collection of *Ulva*

We collected *Ulva* spp. monthly from June to October 2013 at several publically accessible bloom-forming sites in Narragansett Bay, RI, including Chepiwanoxet, Sandy Point, Oakland Beach, Oakland Beach Cove, and Warwick City Park. We chose these sites to represent a range of typical *Ulva* spp. bloom intensity, with Oakland Beach Cove and Warwick City Park as high bloom sites, while Chepiwanoxet, Sandy Point and Oakland Beach as low bloom sites (Thornber, unpublished data). At each site, on each sampling date, we haphazardly collected individuals by hand from the shallow subtidal zone, put them in a plastic bag, and brought them back to the lab. We selected a minimum of 16 individuals and maximum of 40 individuals on each sampling date. Later, we identified *U*. *compressa* and *U*. *rigida* to the species level by microscopic examination and only used individuals with clear cellular characteristics based on the current molecular analyses of *Ulva* in Narragansett Bay [45]. A recent study by Mao et al. 2014 discovered the presence of *U*. *laetevirens* in Long Island Sound [46]. Since there are morphological similarities between *U*. *rigida* and *U*. *laetevirens*, we recognize the potential for species misidentification, however slight, in our study. Overall, we collected and analyzed 282 total *Ulva* individuals: 150 *U*. *compressa* and 132 *U*. *rigida* (S1 Table). Both species were collected at all sites, with a minimum of 10 individuals of each species at each site. Due to the nature of sampling and length of time necessary for preparing flow cytometry samples (which limited our ability to collect larger sample sizes), we present and analyze our data here in terms of the overall relative abundance of each *Ulva* species during the peak bloom-forming season at each site. However, we use collection date and month as covariates in building our logistic regression models for predicting the relative abundance of each phase (see Statistical Analysis section). We used sea surface temperature and sea surface salinity data for Greenwich Bay (Site F5) collected daily by the Rhode Island Department of Environmental Management Bay Assessment and Response Team (http://www.narrbay.org/d_projects/buoy/buoydata.htm; S2 Table).

We also determined *Ulva* biomass data from monthly subtidal surveys of the same sites, following the protocol in Guidone [44]. Briefly, at each site, we collected all algae in each of 0.16 m^2^ subtidal quadrats placed 1 m apart along a transect line. All plots were < 2 m deep at mean lower low water (S1 Table).

Prior to thallus destruction for flow cytometry, we took a microscopic photograph at 400X of each individual that was analyzed for ploidy content. Using ImageJ (www.nih.gov), we created an overlying grid on each microscopic photograph, and measured the area of the exposed surface of the first ten cells that were at grid intersection points to assess cell size differences between phases (S3 Table). We examined the upper cell layer, as *U*. *compressa* and *U*. rigida are each two cells thick.

### Flow Cytometry and Ploidy Analysis

We used flow cytometry to determine the relative abundance of gametophytes and sporophytes in *U*. *rigida* and *U*. *compressa*. Based on the C-values (haploid genome sizes) of *U*. *compressa* 0.13 pg [27] and *U*. *rigida* 0.16 pg [27], we used the freshwater unicellular alga *Chlamydomonas reinhardtii* as an external flow cytometry control, with a C-value of 0.12 pg [47]. We specifically selected the cell wall-deficient mutant CC-400 cw15 mt+ as our control (University of Minnesota *Chlamydomonas* Center, chlamycollection.org). The cell wall-deficient mutant was selected to easily rupture the cells and allow the PI/RNase Staining Buffer to reach the nucleus.

We used an enzyme solution developed specifically for efficient production of *Ulva* protoplasts [48], along with a modified version of the LB01 nuclear isolation buffer. Instead of the standard 0.1% v/v concentration for Triton X-100, we modified the buffer to contain a 1% v/v concentration to ensure the nuclei were cleanly isolated (15mM Tris, 2MM EDTA, 0.5mM Spermine tetrahydrochloride, 80mM KCl, 20mM NaCl, 1% vol/vol Tritron x-100, 15mM β-mercaptoethanol) [26].

We were concerned with successful protoplast isolation and not with the exact number of protoplasts obtained, so we chose a qualitative method for isolating protoplasts [48]. We weighed all *Ulva s*amples to 0.50 g wet weight, rinsed with them raw seawater to remove debris and epiphytes, and then thoroughly scrubbed them manually in 20 μm filtered seawater to remove smaller particles. *Ulva* samples were chopped with a razor blade in a large (85 mm x 25 mm) plastic Petri dish for one minute, and then the tissue was transferred into a small (55 mm x 15 mm) Petri dish that contained 5 mL of enzyme solution [48].

Protoplasts were released by placing samples on a shaker at 50 rpm in the dark for two hours at room temperature (~21°C), then filtered with a 30μm nylon mesh into a 5mL polypropylene tube and spun for five minutes at 120 x g at 4°C. A total of 2mL of supernatant was then removed and replaced with 2mL of sterile filtered seawater. Centrifugation with subsequent replacement of fluid was repeated twice, and after the last round of centrifugation, all supernatant was removed and replaced with 1mL of sterile filtered seawater. We observed successful protoplast isolation via microscopic examination at 400X. In preparation for the flow cytometer samples were spun for five minutes at 120 g at 4°C, the supernatant was removed, and samples were kept refrigerated or on ice.

**Fig 2:**
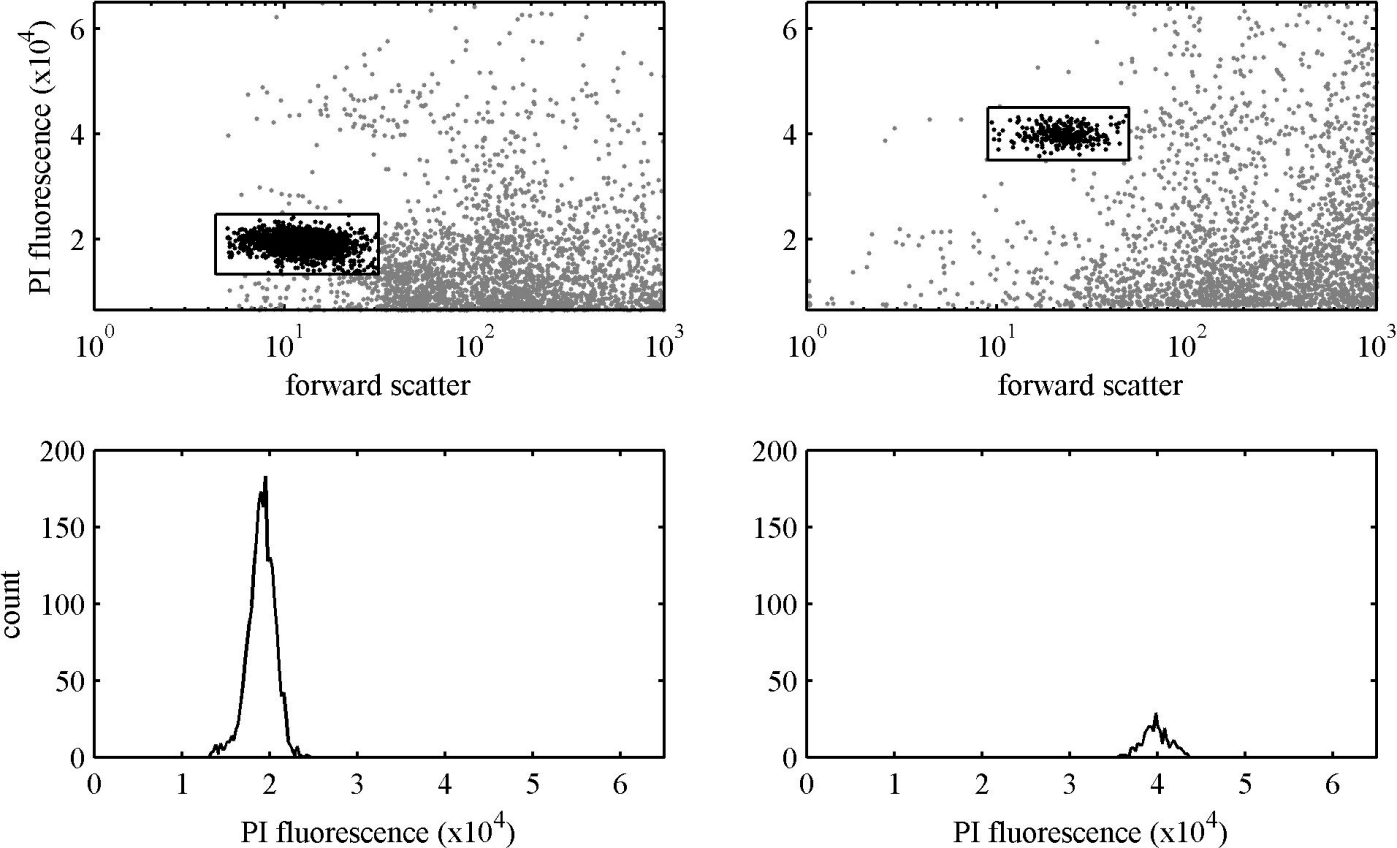
Flow cytometry graphs. The graphs on the left (A, C) represent an *U*. *compressa* gametophyte, while the two graphs on the right (B, D) represent an *U*. *compressa* sporophyte. Fig 2A and 2B show the forward scatter by fluorescence, while Fig 2C and 2D represent the count of nuclei from 20,000 events. The sporophyte (B, D) has twice the fluorescence as the gametophyte (A, C), with the gametophyte mean fluorescence near 19,000 and the sporophyte mean fluorescence near 38,000.

To liberate the nuclei, we added 1 mL of modified LB01 nuclear buffer kept on ice to each sample, vortexed and tapped the tube occasionally for eight minutes, and then added 0.5mL of PI/RNase Staining Buffer (BD Science). After five minutes the samples were run on a BD Influx flow cytometer at the RI EPSCoR Marine Life Sciences Facility on the University of Rhode Island's Narragansett Bay Campus. This machine was optimized for marine applications and is equipped with three lasers (355 nm, 488 nm, and 561 nm). We used a green (532 nm) or a blue (488 nm) laser and quantified fluorescence at 610 nm (20 nm bandwidth) on a linear scale. Since sporophytes have twice the amount of genetic material as gametophytes, sporophytes have twice the amount of fluorescence as gametophytes (Fig 2). To measure the spread of the distribution of the data we used the coefficient of variation (CV), which is the standard deviation expressed as a percentage of the population mean. The CV was calculated from replicate counts of the same prep from one thallus; our CV values ranged from 3–8%. This range is due to the small genome size and the predilection of PI to bind to remaining cell wall polysaccharides from the extraction of *Ulva* protoplasts, which makes obtaining CV values less than 3% challenging [49, 50].

### Growth Experiments

We assessed growth rates of gametophytes and sporophytes of *U*. *rigida* and *U*. *compressa* in outdoor flow-through ambient temperature seawater tanks on the University of Rhode Island’s Narragansett Bay campus. We collected healthy *Ulva* individuals from the shallow subtidal zone in Greenwich Bay in the summer of 2013. In total, we used 90 *U*. *compressa* individuals (62 sporophyte and 28 gametophyte) and 61 *U*. *rigida* individuals (38 sporophyte and 23 gametophyte) for this analysis. We conducted growth experiments in June, July, and August to assess differences in growth over the peak bloom-forming months (S4 Table).

In the lab, we determined the species identity of each specimen via microscopic examination. We then spun individuals 20 times in a salad spinner prior to separating 1.0 g from the thallus. We placed one 1.0g *Ulva* individual in each 2.5 L bucket with mesh sides; after 14 days, all growth experiments concluded and the *Ulva* was re-weighed. For each month, we had a sample size of at least five (up to a maximum of 36) individuals of each phase of each species, except for *U*. *rigida* sporophytes in August, when we only had three individuals. All *Ulva* were spun 20 times in a salad spinner prior to each weighing on a digital scale to ensure consistent mass, and all individuals were analyzed using flow cytometry for ploidy content (see above).

### Statistical Analyses

To assess ploidy ratios in field populations of *U*. *compressa* and *U*. *rigida*, we used a χ^2^ analysis to determine if the relative abundances of each species, at each site, were significantly different from the null model hypothesis. We then assessed the relationship of several variables (site, species, salinity, temperature, month of collection, date of collection, total *Ulva* biomass, total algal biomass, total *Ulva* biomass) to the ploidy ratio, using a logistic regression model with a binomial response variable (gametophyte vs. sporophyte). We selected the model with the highest AIC as it best explained the distribution of gametophytes and sporophytes in Greenwich Bay (S1 Text).

The AIC measures the relative quality of a statistical model, taking into consideration the number of parameters and the information lost with the model. Model coefficient estimate values predict the odds ratio of gametophytes and sporophytes in the population. The model has a binomial response variable with sporophytes chosen as success and gametophytes as failure. Therefore, negative estimate values are associated with higher proportions of gametophytes while positive estimate values are associated with higher proportions of sporophytes.

Based on the results for the logistic regression model described above, we then selected the three significant continuous variables (salinity, salinity two weeks prior to specimen collection, and total *Ulva* biomass) and analyzed each individually in separate models for representation in graphical models. Data analyses were conducted in R [51, 52] and JMP (JMP^®^, Version 10. SAS Institute Inc., Cary, NC, 1989–2013).

Relative growth data were analyzed with a two way fixed factor ANOVA to measure differences across ploidy levels and months. Cell sizes were compared between gametophytes and sporophytes for each species using t-tests with unequal variances in JMP. All data were checked for statistical test assumptions and transformed where appropriate prior to analysis.

### Ethics Statement

All research was conducted on public beaches in Rhode Island. No specific permits were obtained for this research, as the Rhode Island state constitution guarantees its citizens the right collect seaweed from public beaches [53]. The study did not involve any endangered or protected species or any protected locations.

## Results

### Ploidy

**Fig 3:**
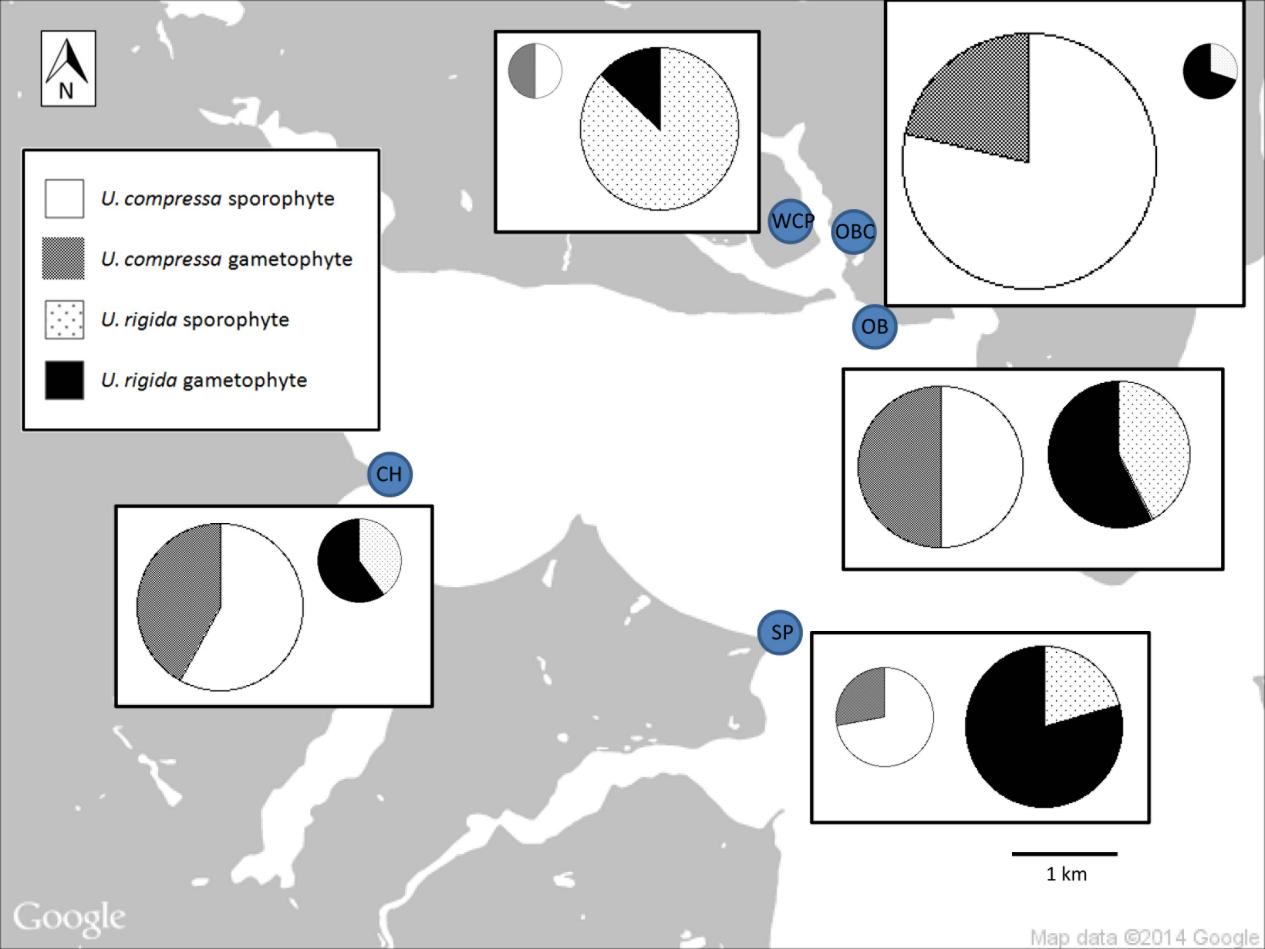
Map of Greenwich Bay (a subset of Narragansett Bay, Rhode Island). This figure shows the relative proportion of gametophytes and sporophytes present at five sites in Greenwich Bay during the 2013 bloom-forming season. The sites are Warwick City Park (WCP), Oakland Beach Cove (OBC), Oakland Beach (OB), Sandy Point (SP), and Chepiwanoxet (CH). Pie chart sizes represent the relative number of individuals sampled.

We found both gametophytes and sporophytes of each species present at each of the sampling location sites (S1 Table). There were significant differences among the relative ploidy levels at each site (Fig 3), compared to the null model prediction of √2 gametophytes to 1 sporophyte (χ^2^likelihood test, Table 1). *U*. *compressa* in Oakland Beach Cove (OBC) and Sandy Point (SP) differed from this null prediction with a relative overabundance of sporophytes. *U*. *rigida* in Warwick City Park (WCP) and Sandy Point (SP) differed from the null prediction with a relative overabundance of sporophytes in WCP and dominance of gametophytes in SP.

**Table 1:** Testing against the null model prediction of √2 gametophytes: 1 sporophyte by site and species. The overabundant phase column indicates which phase was more abundant than predicted by the null model. Numbers in bold indicate significant (<0.05 values).

Site	Species	χ^2^	Sample Size	Overabundant Phase	Prob. > χ^2^
Chepiwanoxet	*U*. *compressa*	3.651	31	Expected	0.056
Oakland Beach	*U*. *compressa*	0.988	30	Expected	0.320
Oakland Beach Cove	*U*. *compressa*	27.877	47	Sporophyte	**<0.001**
Sandy Point	*U*. *compressa*	7.188	18	Sporophyte	**0.007**
Warwick City Park	*U*. *compressa*	0.329	10	Expected	0.566
Chepiwanoxet	*U*. *rigida*	0.006	15	Expected	0.937
Oakland Beach	*U*. *rigida*	0.018	26	Expected	0.892
Oakland Beach Cove	*U*. *rigida*	0.519	10	Expected	0.471
Sandy Point	*U*. *rigida*	5.401	29	Gametophyte	**0.020**
Warwick City Park	*U*. *rigida*	27.024	30	Sporophyte	**<0.001**

Based on AIC values, the strongest predictive model for ploidy relative abundance included the variables species, site, salinity at time of sampling, and total *Ulva* biomass (Table 2; S2 Table; S1 Text) and not temperature, month of sampling, date of sampling, or total algal biomass. While salinity measurements with a time lag of two weeks prior were significant, they were not included in the model with the strongest AIC.

**Table 2:** Table for the best-fit logistic regression with a binomial distribution and ploidy as the sindependent variable. Model follows the form
$$\text{logit}(\hat{y})=\beta_{0}+\beta_{1}x_{1}+\beta_{2}x_{2}+\ldots$$(1)
(e.g. -2.480–1.130 * *U*. *rigida*– 0.946 * Oakland Beach + …). Numbers in bold indicate significant (< 0.05 values).

Coefficient	Estimate	Std. Error	z value	Prob.(>|z|)
Intercept	-2.480	0.953	-2.603	**0.009**
*U*. *rigida*	-1.130	0.629	-1.798	0.072
Oakland Beach	-0.956	0.579	-1.650	0.099
Oakland Beach Cove	0.684	0.523	1.309	0.191
Sandy Point	-0.547	0.683	-0.801	0.423
Warwick City Park	-0.688	0.766	-0.898	0.369
Salinity	0.075	0.019	3.923	**<0.001**
*Ulva* biomass	0.088	0.033	2.667	**0.008**
Oakland Beach*U. *rigida*	0.503	0.860	0.585	0.559
Oakland Beach Cove*U. *rigida*	-0.118	0.940	-0.125	0.900
Sandy Point*U. *rigida*	-0.794	0.946	-0.839	0.402
Warwick City Park*U. *rigida*	2.891	1.051	2.750	**0.006**

**Fig 4:**
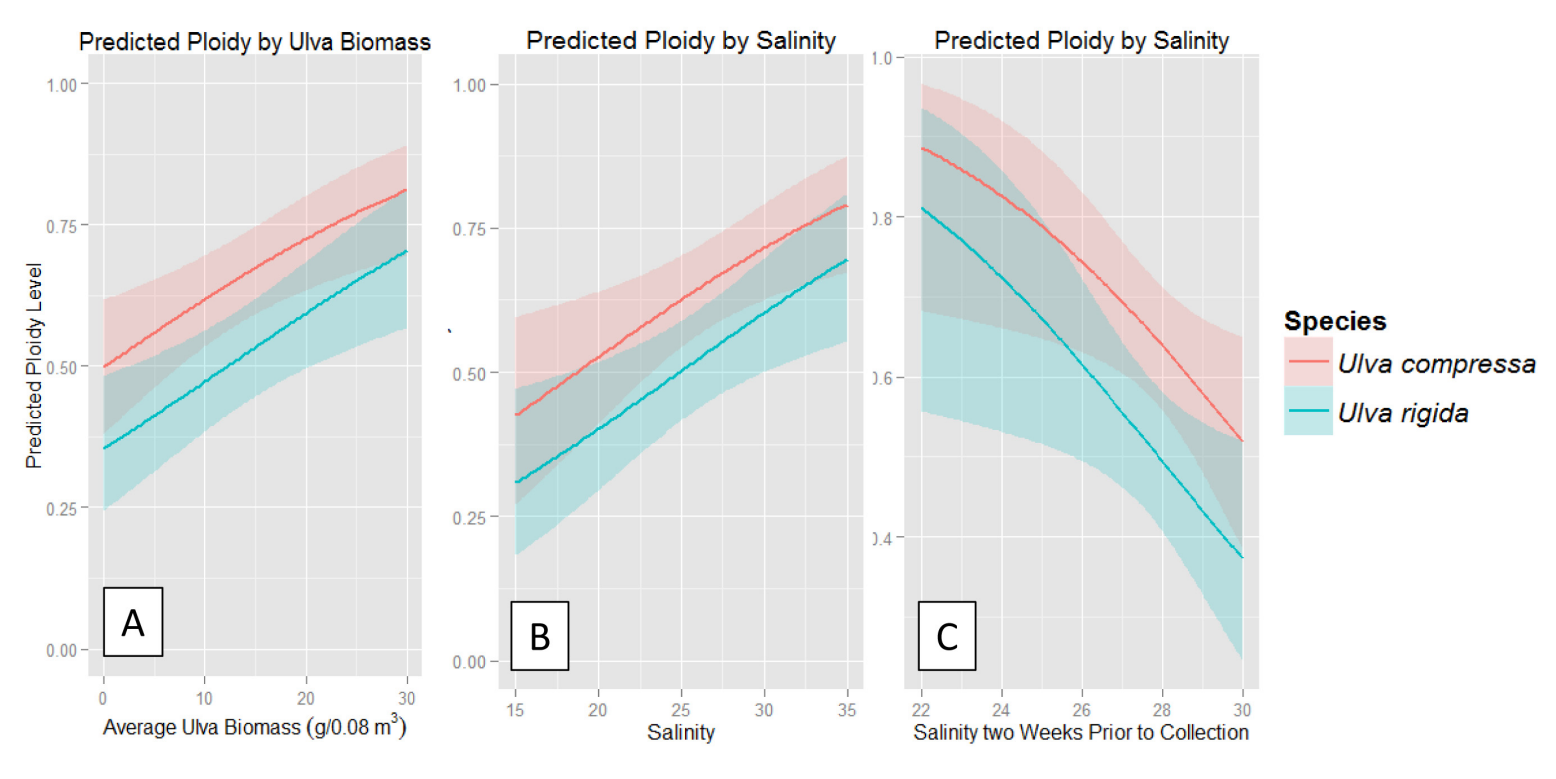
The predicted probability of an individual being a gametophyte or sporophyte under different environmental conditions based on the model estimate. Sporophytes are represented by one, while gametophytes are represented by zero. A value of one indicates 100 percent sporophyte abundance. Variables measured include *Ulva* biomass (g/0.08 m^3^) present at time of collection (4A), surface salinity values from the date collection (4B) and from two weeks prior to collection (4C). The upper line is *U*. *compressa* and the lower line is *U*. *rigida*. The shading indicates 95% confidence intervals.

When we analyzed the significant continuous variables individually for their correlation to ploidy ratios, we found that the relative abundance of sporophytes was positively correlated with higher *Ulva* biomass at the time of collection (Fig 4A; χ^2^_3_ = 16.10, p<0.01). We found increasing proportions of *Ulva* sporophytes at higher salinities at the date of sampling for both species (Fig 4B; χ^2^_3_ = 13.36, p<0.01). Interestingly, salinity measurements with a time lag of two weeks prior yielded significantly increasing proportions of *Ulva* gametophytes at higher salinities (Fig 4C; χ^2^_3_ = 10.54, p = 0.01) for both species.

### Growth

**Fig 5:**
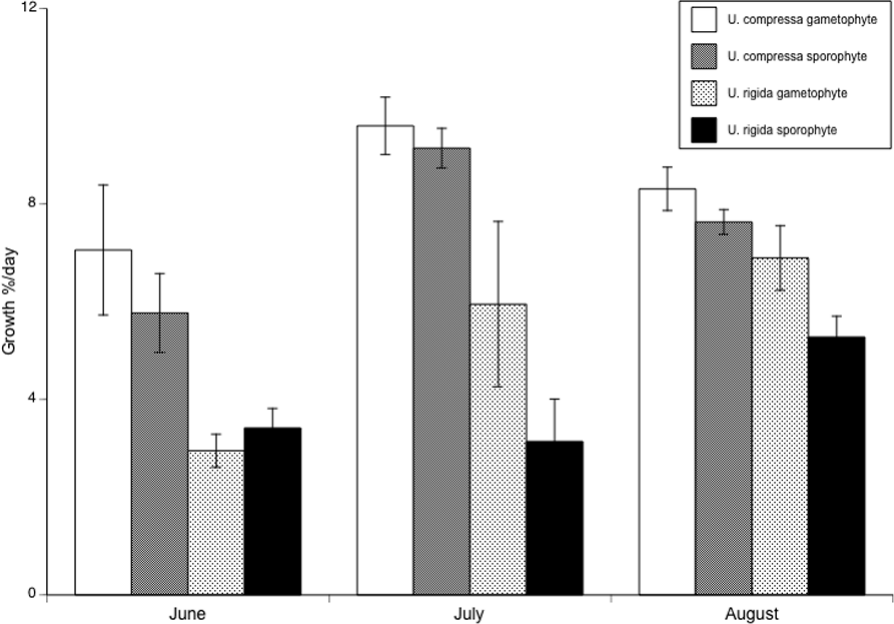
Growth of *U*. *rigida* and *U*. *compressa* gametophytes and sporophytes. There are no significant differences in relative growth rate between ploidy levels in either species. Data are means ± one standard error.

We found no significant differences in relative growth rate between phases for either species, (Fig 5; *U*. *compressa*, F_1,84_ = 1.18, p = 0.28; *U*. *rigida*, F_1,54_ = 0.16, p = 0.69; S4 Table) but we did find differences in growth rate across months (*U*. *compressa*, F_2,84_ = 9.88, p<0.0001; *U*. *rigida*, F_2,54_ = 4.14, p = 0.02). There was no significant interaction between month and ploidy for either species (*U*. *compressa*, F_2,84_ = 0.18, p = 0.83; *U*. *rigida*, F_2,54_ = 1.88, p = 0.16). *U*. *compressa* had a significantly higher growth rate in July than in June or August (post-hoc Tukey-Kramer, F_2,87_ = 11.72, p<0.0001), with a mean relative growth rate (%/day) June = 6.27, July = 9.25, and August = 7.91. *U*. *rigida* also had significantly higher relative growth rate in August vs. June or July (post-hoc Tukey-Kramer, F_2,57_ = 6.73, p = 0.0024), with a mean growth rate (g/day) in June = 3.31, July = 3.94, and August = 6.52.

### Cell Area

**Fig 6:**
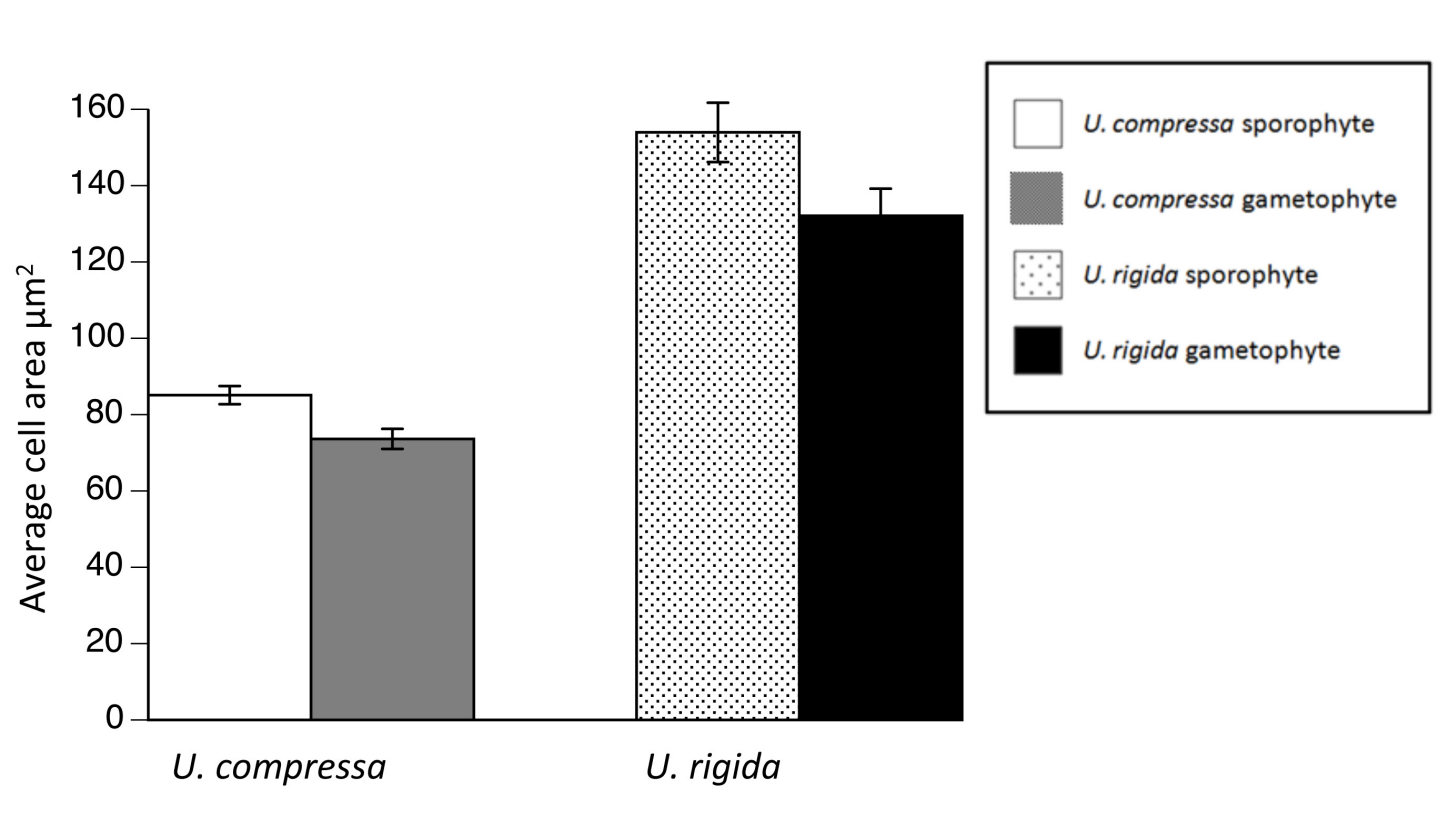
Cell area by species and ploidy for *U*. *compressa* and *U*. *rigida*. Both species have significantly larger sporophyte cell area then gametophyte cell area. Data are means ± one standard error.

*U*. *compressa* sporophytes (mean area = 85.10 ± 2.38 μm^2^) had a larger cell area than gametophytes (mean area = 73.63 ± 3.88 μm^2^; t_60_ = -2.63, p = 0.01; S3 Table). *U*. *rigida* sporophytes (mean area = 153.95 ± 7.77 μm^2^) also had a larger cell area than gametophytes (mean area = 132.12 ± 7.08 μm^2^; t_60_ = -1.98, p = 0.05; Fig 6).

## Discussion

### Ploidy Distribution

Our data indicate that both phases are present for both *U*. *compressa* and *U*. *rigida* throughout the peak bloom-forming season, and that relative phase abundance is correlated with both abiotic and biotic factors. We found a high variability among sites in ploidy ratio among sites, with some sites matching the null model prediction of relative abundance, while others exhibited a significant overabundance of gametophytes or sporophytes. These deviations could be due to ecological differences among phases, environmental differences among sites, and/or temporal differences in life cycle dynamics among sites. Sandy Point, which differed from the null hypothesis for both species, is a more exposed site and experiences more water mixing than the other sites [54]. However, as *U*. *compressa* had an overabundance of sporophytes and *U*. *rigida* had an overabundance of gametophytes at this site, the relative impacts of environmental factors are challenging to assess and may represent specific environmental factors unique to each species. Warwick City Park and Oakland Beach Cove, which differed from the null hypothesis in *U*. *compressa* and *U*. *rigida* respectively, are more sheltered sites and experience less water mixing [54].

We found a significant correlation of physical and biological factors on the relative abundance of gametophytes and sporophytes in our study system (Table 2, Fig 4). In this study system, low salinities are typically a result of increased freshwater flow from rivers caused by storms. In Narragansett Bay, increased flow in rivers yields higher concentrations of dissolved inorganic nitrogen and phosphorus [55]. Therefore, although nutrient data are not available for our sampling period, low salinities can be used as a proxy for increased nutrients. Lower salinities from the date of sample collection were correlated with higher relative levels of gametophytes, while lower salinities from two weeks prior to specimen collection were correlated with more sporophytes (Fig 4). This shift in ploidy ratios may be due to several factors, such as salinity tolerance, positive response to nutrient availability from one phase over the other, or a shift to asexual reproduction [56]. While it is unlikely that a reproductive event would result in the presence of new adults after only two weeks [57], lower salinities may trigger more rapid growth of one phase from a microscopic to a macroscopic size [35]. Due to the biphasic life cycle, increased nutrients may either impact mortality and/or fecundity rates of either phase [40, 58], with differential effects on the relative balance of phases. In addition, vegetative fragmentation of mature blades, germination of unfused gametes, and/or asexual production of diploid spores by sporophytes may impact the ploidy ratio [18].

We also found a positive correlation between the relative abundance of sporophytes for total *Ulva* biomass for both species. This may be a byproduct of the positive correlation of temperature with bloom abundance [59, 60] and growth rates [44], although we found no impact of temperature on the relative abundance of gametophytes and sporophytes in this study.

Previous studies have found a seasonal dominance of one ploidy phase [30] or a long term (11–20 month) non-seasonal cyclic dominance [19], or no seasonal trend [61]. As our sampling was limited to the bloom forming season, a cycling trend in ploidy for *U*. *compressa* and/or *U*. *rigida* could exist. However, due to the scarcity of *Ulva* specimens during non bloom forming periods [43], this would be challenging to assess.

### Growth and Cell Area

We did not find any significant differences in growth rates of adult gametophytes and sporophytes of either species, but this does not preclude the possibility of differences at the germling stage [35]. In addition, growth rates can vary based on nutrient levels [62]; as nutrient levels shift in Narragansett Bay over seasonal cycles [63, 64], differences in *Ulva* growth rates between phases may emerge.

Based on our cell area data, future studies of *U*. *compressa* and *U*. *rigida* life cycle dynamics may be much more rapid. Individuals can be predicted as gametophytes or sporophytes based on their cell area, with a subset confirmed using ploidy analysis. This would increase the ability to have larger sample sizes and more rapid assessment.

Differences in *U*. *compressa* and *U*. *rigida* cell areas between phases may impact the surface area to volume ratio, allowing for faster uptake of nutrients in smaller cells [40]. This is especially relevant in single-celled spores, gametes, and small juveniles, and may impact *Ulva* individuals in their early growth stages. *U*. *rigida* zoospores are 9–15 μm x 5–10 μm while gametes are 7–11 μm x 4–6 μm [65]. Since gametes are smaller than zoospores, they may have a survival differential in their ability for nutrient uptake and storage capacity. There may also be other ecological differences between either phases across their lifespan, such as susceptibility to herbivores, light tolerance, salinity tolerances, and temperature optima [23, 29, 34, 66], that may explain differences in ploidy ratios.

### Flow Cytometry Method

We designed our flow cytometry ploidy analysis methods from similar analyses in higher plants [26, 49, 67, 68], which has been successful for other macroalgal studies [27]. We first attempted chopping *Ulva* tissues with a razor blade in the presence of a nuclear isolation buffer to obtain isolated nuclei (essentially removing our protoplast isolation step). This method, which is successful in higher plants for flow cytometric analysis [69], was unsuccessful for *Ulva*. The number of nuclei obtained was small and contaminated with other materials, likely organelle genomes and bacteria [70]. In addition, *Ulva* has high concentrations of anionic polysaccharides in its cell walls [71] which can interfere with obtaining a sufficient number of nuclei by binding to the positively charged nuleus, inhibiting the propidium iodide from attaching. Given these constraints, protoplast isolation was necessary to obtain sufficient numbers of nuclei for flow cytometry analyses [50], which is successful yet time consuming [48], thus limiting our abilities to obtain larger sample sizes.

## Supporting Information

S1 Table Ploidy biomass physical data for R. (XLSX)

S2 Table Physical factor data. (XLSX)

S3 Table Cell size data. (XLSX)

S4 Table Growth data. (XLSX)

S1 Text R code for ploidy analyses and model predictions. (DOCX)
