# Supplementary material for: Ploidy Distribution of the Harmful Bloom Forming Macroalgae Ulva spp. in Narragansett Bay, Rhode Island, USA, Using Flow Cytometry Methods
Source: PLoS One. 2016 Feb 26;11(2):e0149182. doi: 10.1371/journal.pone.0149182 (PMC4769327; doi:10.1371/journal.pone.0149182)
Supplement: S1 Text — (DOCX) [file pone.0149182.s005.docx]

ulva = read.table("R Data.csv", header=T, sep=",", na.strings=c("."," "))

head(ulva)

attach(ulva)

sapply(ulva, class)

ulva$Dead.scale = as.factor(ulva$Dead.scale)

ulva$Ploidy = as.character(ulva$Ploidy)

ulva$Site = as.character(Site)

ulva$Species = as.character(Species)

ulva$Date.as.Number = as.numeric(Date.as.Number)

# Packages I need

library(lattice)

library(stats)

library(MASS)

library(plyr)

library(graphics)

library(bbmle)

library(data.table)

library(emdbook)

library(aod)

library(ggplot2)

# for this analysis, need numbers between 0 and 1

# change 2s to 1s and 1s to 0s

ulva3 = ulva

ulva3$Ploidy[ulva3$Ploidy == "1"] <- "0"

ulva3$Ploidy[ulva3$Ploidy == "2"] <- "1"

sapply(ulva3, class)

ulva3$Ploidy <- as.factor(ulva3$Ploidy)

#run logit models

mylogit3 <- glm(Ploidy ~ Salinity.Greenwich.Bay

+ Temp.Greenwich.Bay + Species,

data = ulva3, family = "binomial")

summary(mylogit3)

library(aod)

library(ggplot2)

## using means

newdata1 <- with(ulva3, data.frame(Salinity.Greenwich.Bay = mean(Salinity.Greenwich.Bay),

Temp.Greenwich.Bay = mean(Temp.Greenwich.Bay), Species = c("Ulva compressa", "Ulva rigida")))

## view data frame

newdata1

# adding column

newdata1$SpeciesP <- predict(mylogit3, newdata = newdata1, type = "response")

newdata1

sum(newdata1$SpeciesP) #get 0.8348873, but doesn't need to be 1

newdata2 <- with(ulva3, data.frame(Salinity.Greenwich.Bay = rep(seq(from = 15, to = 35, length.out = 100), 2)

, Temp.Greenwich.Bay = mean(Temp.Greenwich.Bay), Species = rep(c("Ulva compressa", "Ulva rigida"), each = 100)))

newdata3 <- cbind(newdata2, predict(mylogit3, newdata = newdata2, type = "link",

se = TRUE))

newdata3 <- within(newdata3, {

PredictedProb <- plogis(fit)

LL <- plogis(fit - (1.96 * se.fit)) #where does the 1.96 come from?

UL <- plogis(fit + (1.96 * se.fit))

})

## view first few rows of final dataset

head(newdata3)

#graph salinity vs predicted ploidy probability

h <- ggplot(newdata3, aes(x = Salinity.Greenwich.Bay, y = PredictedProb))

h + geom_ribbon(aes(ymin = LL, ymax = UL, fill=newdata3$Species),

alpha = 0.2) + geom_line(aes(colour = Species),

size = 1) +

ylim(0,1) +

xlab("Salinity") +

ylab("Predicted Ploidy Level") +

ggtitle("Predicted Ploidy by Salinity") +

labs(fill="Species") +

guides(fill = guide_legend(keywidth = 2, keyheight = 2)) +

theme(legend.text = element_text(size = 16, face = "italic")) +

theme(legend.title = element_text(size=16))

# find test statistic

with(mylogit3, null.deviance - deviance)

# DF

with(mylogit3, df.null - df.residual)

#p value

with(mylogit3, pchisq(null.deviance - deviance, df.null - df.residual, lower.tail = FALSE))

#log likelihood

#likelihood ratio test (the deviance residual is -2*log likelihood).

logLik(mylogit3)

head(ulva3)

mylogit4 <- glm(Ploidy ~ Salinity.two.weeks.before

+ Temp.Greenwich.Bay + Species,

data = ulva3, family = "binomial")

summary(mylogit4)

## using means

newdata1 <- with(ulva3, data.frame(Salinity.two.weeks.before = mean(Salinity.two.weeks.before),

Temp.Greenwich.Bay = mean(Temp.Greenwich.Bay), Species = c("Ulva compressa", "Ulva rigida")))

## view data frame

newdata1

newdata1$SpeciesP <- predict(mylogit4, newdata = newdata1, type = "response")

newdata1

sum(newdata1$SpeciesP) #get 0.8348873, but doesn't need to be 1

newdata2 <- with(ulva3, data.frame(Salinity.two.weeks.before = rep(seq(from = 22, to = 30, length.out = 100), 2)

, Temp.Greenwich.Bay = mean(Temp.Greenwich.Bay), Species = rep(c("Ulva compressa", "Ulva rigida"), each = 100)))

newdata2

newdata3 <- cbind(newdata2, predict(mylogit4, newdata = newdata2, type = "link",

se = TRUE))

newdata3 <- within(newdata3, {

PredictedProb <- plogis(fit)

LL <- plogis(fit - (1.96 * se.fit)) #where does the 1.96 come from?

UL <- plogis(fit + (1.96 * se.fit))

})

## view first few rows of final dataset

head(newdata3)

#graph salinity two weeks before vs predicted ploidy probability

h <- ggplot(newdata3, aes(x = Salinity.two.weeks.before, y = PredictedProb))

h + geom_ribbon(aes(ymin = LL, ymax = UL, fill=newdata3$Species),

alpha = 0.2) + geom_line(aes(colour = Species),

size = 1) +

xlab("Salinity two Weeks Prior to Collection") +

ylab("Predicted Ploidy Level") +

ggtitle("Predicted Ploidy by Salinity") +

labs(fill="Species") +

guides(fill = guide_legend(keywidth = 2, keyheight = 2)) +

theme(legend.text = element_text(size = 16, face = "italic")) +

theme(legend.title = element_text(size=16))

# find test statistic

with(mylogit4, null.deviance - deviance)

# DF

with(mylogit4, df.null - df.residual)

#p value

with(mylogit4, pchisq(null.deviance - deviance, df.null - df.residual, lower.tail = FALSE))

#log likelihood

#likelihood ratio test (the deviance residual is -2*log likelihood).

logLik(mylogit4)

head(ulva3)

mylogit5 <- glm(Ploidy ~ Average.Monthly.Ulva.Biomass

+ Temp.Greenwich.Bay + Species,

data = ulva3, family = "binomial")

summary(mylogit5)

## using means

newdata1 <- with(ulva3, data.frame(Average.Monthly.Ulva.Biomass = mean(Average.Monthly.Ulva.Biomass),

Temp.Greenwich.Bay = mean(Temp.Greenwich.Bay), Species = c("Ulva compressa", "Ulva rigida")))

## view data frame

newdata1

newdata1$SpeciesP <- predict(mylogit5, newdata = newdata1, type = "response")

newdata1

sum(newdata1$SpeciesP) #get 0.8348873, but doesn't need to be 1

summary(Average.Monthly.Ulva.Biomass)

newdata2 <- with(ulva3, data.frame(Average.Monthly.Ulva.Biomass = rep(seq(from = 0, to = 30, length.out = 100), 2)

, Temp.Greenwich.Bay = mean(Temp.Greenwich.Bay), Species = rep(c("Ulva compressa", "Ulva rigida"), each = 100)))

newdata2

newdata3 <- cbind(newdata2, predict(mylogit5, newdata = newdata2, type = "link",

se = TRUE))

newdata3 <- within(newdata3, {

PredictedProb <- plogis(fit)

LL <- plogis(fit - (1.96 * se.fit)) #where does the 1.96 come from?

UL <- plogis(fit + (1.96 * se.fit))

})

## view first few rows of final dataset

head(newdata3)

#graph ulva biomass vs predicted ploidy probability

h <- ggplot(newdata3, aes(x = Average.Monthly.Ulva.Biomass, y = PredictedProb))

h + geom_ribbon(aes(ymin = LL, ymax = UL, fill=newdata3$Species),

alpha = 0.2) + geom_line(aes(colour = Species),

size = 1) +

ylim(0,1) +

xlab(expression("Average Ulva Biomass"~("g/0.08 m"^"3"))) +

ylab("Predicted Ploidy Level") +

ggtitle("Predicted Ploidy by Ulva Biomass") +

labs(fill="Species") +

guides(fill = guide_legend(keywidth = 2, keyheight = 2)) +

theme(legend.text = element_text(size = 16, face = "italic")) +

theme(legend.title = element_text(size=16))

# find test statistic

with(mylogit5, null.deviance - deviance)

# DF

with(mylogit5, df.null - df.residual)

#p value

with(mylogit5, pchisq(null.deviance - deviance, df.null - df.residual, lower.tail = FALSE))

#log likelihood

#likelihood ratio test (the deviance residual is -2*log likelihood).

logLik(mylogit5)

summary(mylogit5)
